# A dataset of tomato fruits images for object detection in the complex lighting environment of plant factories

**DOI:** 10.1016/j.dib.2023.109291

**Published:** 2023-06-03

**Authors:** Zhen-wei Wu, Ming-hao Liu, Cheng-xiu Sun, Xin-fa Wang

**Affiliations:** a School of Information Engineering, Henan Institute of Science and Technology, Xinxiang, China; b College of Mechanical and Electrical Engineering, Xinxiang University, Xinxiang, China; c School of Information Engineering, Zhengzhou Electric Power Technology College, Zhengzhou, China

**Keywords:** Tomato dataset, Fruit object detection, Artificial light plant factory, Computer vision

## Abstract

Plant factories are an advanced form of facility agriculture that enable efficient plant cultivation through controllable environmental conditions, making them highly suitable for the automation and intelligent application of machinery. Tomato cultivation in plant factories has significant economic and agricultural value and can be utilized for various applications such as seedling cultivation, breeding, and genetic engineering. However, manual completion is still required for operations such as detection, counting, and classification of tomato fruits, and the application of machine detection is currently inefficient. Furthermore, research on the automation of tomato harvesting in plant factory environments is limited due to the lack of a suitable dataset. To address this issue, a tomato fruit dataset was constructed for plant factory environments, named as TomatoPlantfactoryDataset, which can be quickly applied to multiple tasks, including the detection of control systems, harvesting robots, yield estimation, and rapid classification and statistics. This dataset features a micro tomato variety and was captured under different artificial lighting conditions, including changes in tomato fruit, complex lighting environment changes, distance changes, occlusion, and blurring. By facilitating the intelligent application of plant factories and the widespread adoption of tomato planting machinery, this dataset can contribute to the detection of intelligent control systems, operation robots, and fruit maturity and yield estimation. The dataset is publicly available for free and can be utilized for research and communication purposes.

**Specifications Table**

**Table:** 

Subject	Agricultural Sciences
Specific subject area	Image Processing, Image Identification, Image classification, object detection, computer vision, artificial intelligence, deep learning and reinforce learning
Type of data	Images, and text files
How data were acquired	Images were captured using two cameras: Canon 80D Digital Single Lens Reflex (DSLR), and iPhone 11 Wide-angle camera. During image capture, camera settings such as aperture, white balance, ISO sensitivity, and focus were set to automatic mode and controlled by predefined programs. Images that were out of focus or unrelated to tomatoes were excluded.
Data format	Raw and analyzed
Description of data collection	Data was collected in an artificial light plant factory laboratory where tomatoes were grown, capturing tomato fruit images under different artificial light cycles from tomato flowering in December 2021 to the presence of a significant number of ripe fruits in February 2022.
Data source location	Artificial Light Plant Factory Laboratory at the Henan Institute of Science and Technology (HIST), located in Xinxiang, China.
Data accessibility	Repository name: Mendeley Data Data identification number: 10.17632/8h3s6jkyff.1 Direct URL to data: http://dx.doi.org/10.17632/8h3s6jkyff.1[1]
Related to research article	X. Wang, Z. Wu, M. Jia, T. Xu, C. Pan, X. Qi, M. Zhao, Lightweight SM-YOLOv5 Tomato Fruit Detection Algorithm for Plant Factory, Sensors. 23 (2023) 3336. 10.3390/s23063336[2]

## Value of the Data

• The dataset has a size of approximately 3GB and includes 520 high-quality images with clear details. These images are captured at two different resolutions: around 12 MP (4032 × 3024) and 25 MP (4000 × 6000). In comparison to existing datasets that typically have around 1 MP (1270 × 720) and lower image quality due to poor sensor performance [3,4], this dataset offers more pixel information and higher imaging quality. The dataset is further enriched by the presence of complex ambient lighting. In the context of advanced facility agriculture, such as artificial light plant factories, the use of artificial lighting creates a complex lighting environment. Additionally, the lighting conditions vary throughout the plant growth cycle, adding to the complexity of the dataset. These variations in complex lighting conditions pose additional challenges for tomato object detection.
• The constructed tomato fruit classification dataset can be used for training deep learning models to apply to various monitoring, prediction, and operation machine tasks. Additionally, the lightweight tomato fruit detection algorithm described in [2] helps to improve the detection efficiency and accuracy of tomato fruits in plant factories, with significantly better performance than traditional methods. The dataset images also provide Ground Truth, which can be used to quickly initiate tomato fruit detection tasks, and new datasets can be collected based on verification results for further research.
• The dataset primarily serves researchers in the field of agricultural engineering, and the dataset can be used to develop tomato fruit detection models and systems to assist in tomato detection through tasks such as developing visual models. In a plant factory environment, growers can use this dataset to improve research efficiency and develop new standards and methods for performing key tasks in plant factories.
• The dataset is a cross-application of modern agriculture and computer vision disciplines aimed at improving the use of key technologies in agriculture. The dataset can also be used to evaluate the performance of deep learning object detection models and validate their benchmark performance for application in other fields. In addition, the dataset can be used to train weight parameters and transfer them for application in related scenario datasets with similar tasks.

## 1 Objective

While researching and designing detection algorithms and intelligent harvesting machinery for tomato cultivation in plant factories, we have identified a lack of suitable and standardized datasets for training and testing deep learning methods, particularly in the complex lighting conditions of artificial light plant factories. Intelligent harvesting machinery often relies on embedded processors with limited computational power, necessitating the use of lightweight and high-precision detection methods that take into account small target tomatoes and complex lighting environments. We have reviewed current detection methods and proposed a lightweight small-target tomato detection method named SM-YOLOv5 (Small-Mobilenet-Yolov5)[2]. Our research dataset provides essential data support for our detection method, enabling us to rapidly test models and validate improvements against benchmark datasets.

## 2 Data Description

The present dataset comprises 520 images obtained by utilizing an EOS 80D DSLR camera and an iPhone 11 Wide-angle camera, wherein each image has two diverse resolutions of 6000 × 4000 and 4032 × 3024. All images are encoded in standard JPG format and have been labeled via labelImg^1^ software, which generates annotations in Pascal VOC XML [5] format, followed by their conversion into YOLO [6] format. These formats are extensively employed for the purpose of object detection tasks, rendering them convenient for performance evaluation and usage in varied algorithmic research pursuits. Elaborate descriptions of the dataset instances are presented in Table 1.

**Table 1:** Statistical classification of fruit instances in the dataset.

	Number
Green	5996
Red	3116
Total	9112

**Fig. 1:**
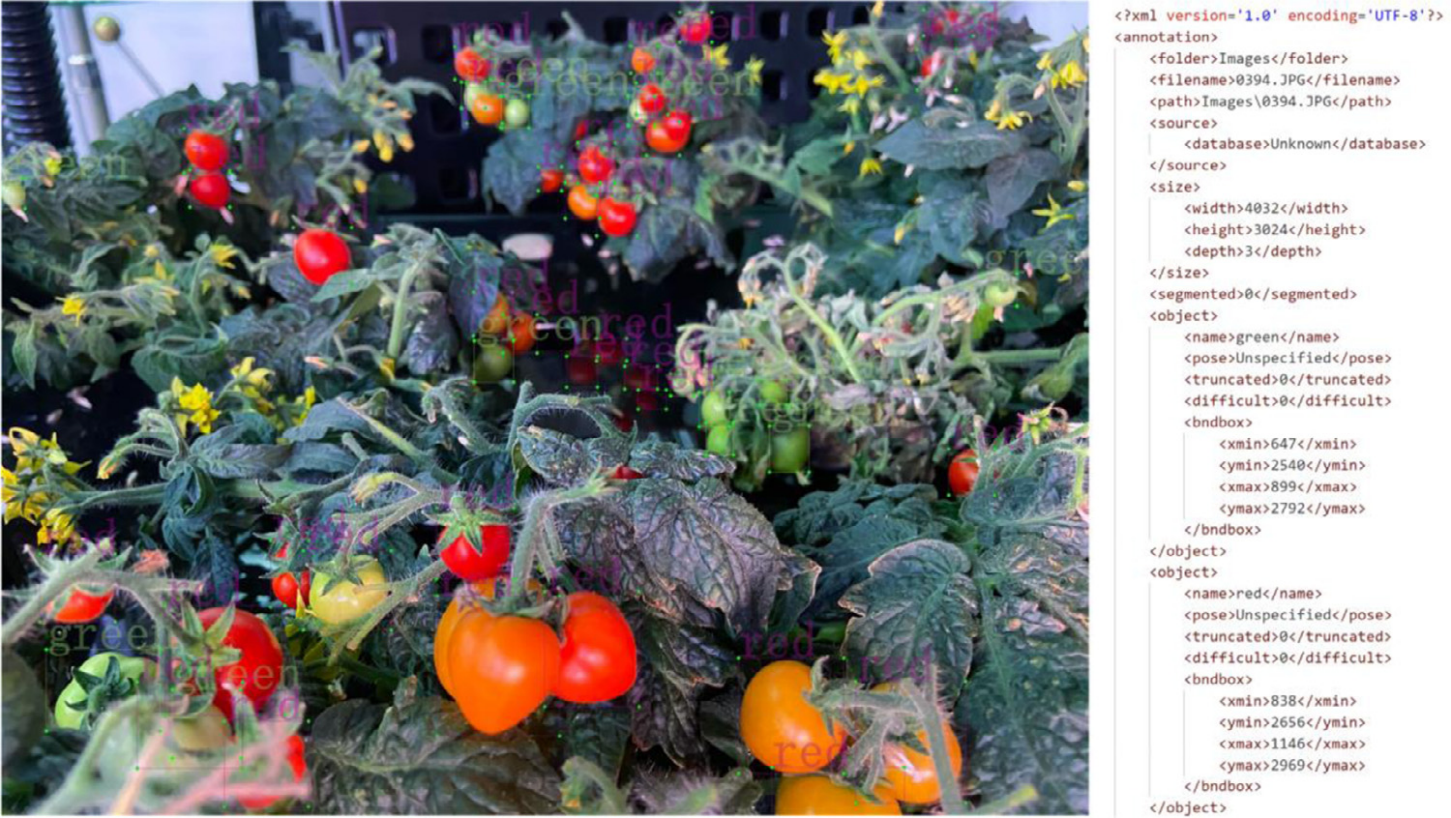
Tomato fruit labeling and Pascal VOC XML annotation source file

**Fig. 2:**
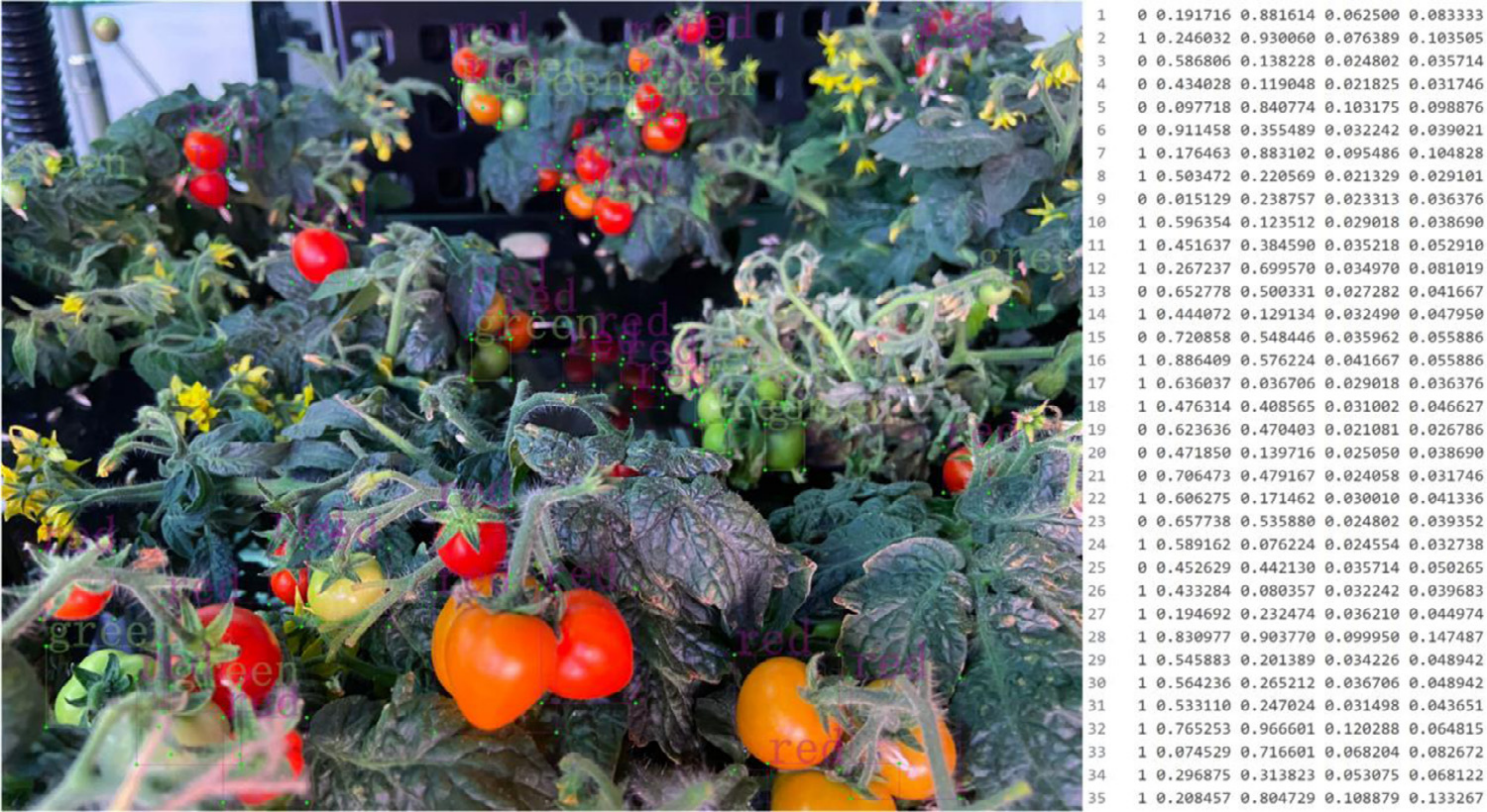
Tomato fruit labeling and YOLO annotation source file

The present dataset depicts tomato fruits categorized into two types, namely green fruits and red fruits, signifying various stages of fruit growth, as illustrated in Fig. 1. The annotation process was performed using Pascal VOC format, where the label information encompasses the positional and category information (xmin, ymin, xmax, ymax) of each instance of fruit. Additionally, YOLO annotation format was also employed, as depicted in Fig. 2, where the annotation details contain instance coordinates information (cls, x, y, width, and height), which are relative to the image size, thereby not compromising the annotation accuracy during proportional scaling of the image. The YOLO annotation format is extensively used in object detection tasks and facilitates the evaluation of algorithmic performance and its applicability to different algorithms.

The processed dataset is segregated into three distinct directories based on file categories: Images, which incorporates all image files; Annotations, that contains the Pascal VOC XML annotation files; and labels, that encompasses the YOLO format annotation files. Each image file present within the Images folder is accompanied by a corresponding annotation file having the same name in both the Annotations and labels directories, respectively, and aligned with their corresponding annotation formats.

## 3 Experimental Design, Materials and Methods

### 3.1 Image Acquisition

**Fig. 3:**
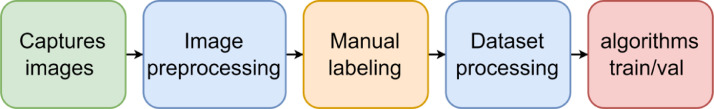
Flowchart of developing the dataset

**Fig. 4:**
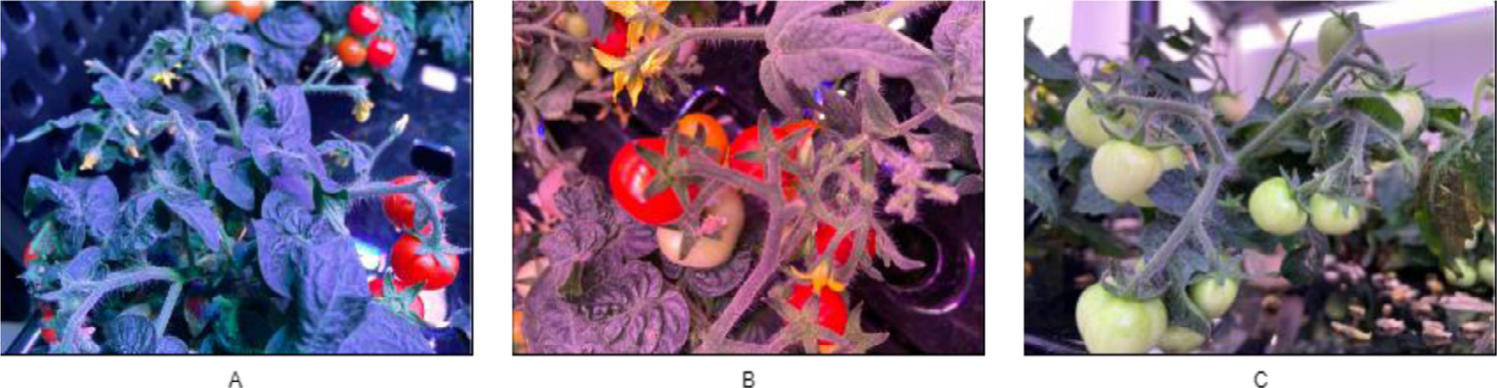
Comparison of supplemental lighting for tomatoes at different growth stages. A, with more blue light, is shown as an overall blue hue in the image; B, with more red light, results in a red hue throughout the image; C, with more white light, shows normal plant colors. The observed variations in lighting conditions are the outcome of dynamic and intelligent control by the plant factory's environmental control system, which adjusts the lighting based on the specific growth stages of the plants. This adaptive control aims to maintain an optimal lighting environment required for plant growth.

Fig. 3 illustrates the process of creating the dataset. The dataset was collected in the fully artificial light plant factory laboratory of Henan Institute of Science and Technology, located in Xinxiang, China. Micro tomato varieties were selected as the experimental objects, and data collection commenced in December 2021 when green tomatoes appeared, and concluded in February 2022 when a substantial amount of red fruits were present. Two cameras were employed to capture images: a Canon 80D DSLR camera for high-definition images (with a resolution of 6000 × 4000 pixels) and an iPhone 11 Wide-angle camera for supplementary images (with a resolution of 4032 × 3024 pixels). As shown in Fig. 4, the light ratio in the artificial light plant factory changes with the growth cycle of tomato plants [7]. Hence, images were taken randomly during different time periods. Moreover, the presence of supplementary lights influences the color of the images, resulting in red, blue, normal, and other color casts.

### 3.2 Image Preprocessing

To mitigate the impact of extraneous data during the processing phase, a uniform nomenclature was adopted for collected images and relevant information in the Exif metadata was expunged.

### 3.3 Manual Image Labelling

All green and red tomato images in this dataset have been manually annotated, ensuring accurate identification by the human eye in the case of displaying the original image at 100% resolution, even in cases of small targets, severe occlusion, or blurriness. Multiple validations have been conducted to minimize errors as much as possible. Annotations were performed using the LabelImg tool to mark the category and location information of instances. The generated Pascal VOC XML annotation files have been preserved and simultaneously converted to the YOLO format to enhance the dataset's diversity and facilitate its application in various object detection models and algorithms.

## Ethics Statement

This study did not conduct experiments involving humans and animals.

## CRediT authorship contribution statement

**Zhen-wei Wu:** Conceptualization, Methodology, Writing – original draft, Investigation, Supervision, Writing – review & editing. **Ming-hao Liu:** Conceptualization, Writing – original draft. **Cheng-xiu Sun:** Conceptualization, Investigation, Funding acquisition. **Xin-fa Wang:** Conceptualization, Methodology, Writing – original draft, Software, Data curation.

## Declaration of Competing Interest

The authors declare that they have no known competing financial interests or personal relationships which have, or could be perceived to have, influenced the work reported in this article.

## Data Availability

TomatoPlantfactoryDataset (Original data) (Mendeley Data).

## Footnotes

1 https://github.com/heartexlabs/labelImg
